# Characterization and possible function of glyceraldehyde-3-phosphate dehydrogenase-spermatogenic protein GAPDHS in mammalian sperm

**DOI:** 10.1186/s12958-015-0008-1

**Published:** 2015-03-08

**Authors:** Hasmik Margaryan, Andriy Dorosh, Jana Capkova, Pavla Manaskova-Postlerova, Anatoly Philimonenko, Pavel Hozak, Jana Peknicova

**Affiliations:** Laboratory of Reproductive Biology, Institute of Biotechnology AS CR, v. v. i, Videnska 1083, 142 20, Prague 4, Czech Republic; Laboratory of Biology of the Cell Nucleus, Institute of Molecular Genetics AS CR, v. v. i, Videnska 1083, 142 20, Prague 4, Czech Republic

**Keywords:** Monoclonal antibodies, Spermatozoa, GAPDHS, Immunolabelling, Transmission electron microscopy, *in vitro* sperm/zona pellucida binding assay

## Abstract

**Background:**

Sperm proteins are important for the sperm cell function in fertilization. Some of them are involved in the binding of sperm to the egg. We characterized the acrosomal sperm protein detected by a monoclonal antibody (MoAb) (Hs-8) that was prepared in our laboratory by immunization of BALB/c mice with human ejaculated sperms and we tested the possible role of this protein in the binding assay.

**Methods:**

Indirect immunofluorescence and immunogold labelling, gel electrophoresis, Western blotting and protein sequencing were used for Hs-8 antigen characterization. Functional analysis of GAPDHS from the sperm acrosome was performed in the boar model using sperm/zona pellucida binding assay.

**Results:**

Monoclonal antibody Hs-8 is an anti-human sperm antibody that cross-reacts with the Hs-8-related protein in spermatozoa of other mammalian species (boar, mouse). In the immunofluorescence test, Hs-8 antibody recognized the protein localized in the acrosomal part of the sperm head and in the principal piece of the sperm flagellum. In immunoblotting test, MoAb Hs-8 labelled a protein of 45 kDa in the extract of human sperm. Sequence analysis identified protein Hs-8 as GAPDHS (glyceraldehyde 3-phosphate dehydrohenase-spermatogenic). For this reason, commercial mouse anti-GAPDHS MoAb was applied in control tests. Both antibodies showed similar staining patterns in immunofluorescence tests, in electron microscopy and in immunoblot analysis. Moreover, both Hs-8 and anti-GAPDHS antibodies blocked sperm/zona pellucida binding.

**Conclusion:**

GAPDHS is a sperm-specific glycolytic enzyme involved in energy production during spermatogenesis and sperm motility; its role in the sperm head is unknown. In this study, we identified the antigen with Hs8 antibody and confirmed its localization in the apical part of the sperm head in addition to the principal piece of the flagellum. In an indirect binding assay, we confirmed the potential role of GAPDHS as a binding protein that is involved in the secondary sperm/oocyte binding.

## Background

Sperm proteins are important for the structure and function of these specific, highly differentiated cells. The function of these proteins turned out to be involved in energy production (23%), transcription, protein synthesis, transport, folding and turnover (23%), cell cycle, apoptosis and oxidative stress (10%), signal transduction (8%), cytoskeleton, flagella and cell movement (10%), cell recognition (7%), metabolism (6%) binding of sperm to the oocyte and other unknown functions (11%) [1-5].

D-Glyceraldehyde-3-phosphate dehydrogenase (GAPDH, EC 1.2.1.12) is a glycolytic enzyme catalysing oxidative phosphorylation of glyceraldehyde-3-phosphate, yielding 1,3-diphosphoglycerate, which is used by phosphoglycerate kinase to produce ATP. In addition, glycolysis results in production of pyruvate, which is a substrate for mitochondria. Therefore, the enzyme plays a significant role in cellular metabolism and energy regulation. In mammals, there are two isoenzymes encoded by two different genes: somatic isoform (GAPDH) and sperm isoform (GAPDHS). GAPDH is present in all tissues of the organism and is localized predominantly in the cell cytoplasm. After breaking of cells, GAPDH is easily extracted with aqueous solutions. The enzyme consists of four identical subunits of 36 kDa. Each subunit of human muscle GAPDH consists of 335 amino acid residues (UniProtKB/Swiss-Prot ID: G3P_HUMAN). The central role in the catalysis is played by the cysteine residue of the active site (Cys 152). The enzyme can be easily affected by different oxidants, resulting in oxidation of the essential cysteine residues with complete loss of the dehydrogenase activity [6-8].

Glyceraldehyde-3-phosphate dehydrogenase-S, GAPDHS, is highly conserved between species, showing 94% identity between rat and mouse and 87% identity between rat and human. Within a particular species, GAPDHS also shows significant sequence similarity to its GAPDH paralog (70%, 71% and 68% for the rat, mouse, and human, respectively). Previous studies of the sperm-specific isoform of the glycolytic enzyme GAPDH – GAPDHS – show a high conservation level of the protein sequence between the two proteins, with the exception of the extra N-terminal part of GAPDHS. This proline-rich part confers a change in biochemical properties of the enzyme. While GAPDH is an abundant cytoplasmic protein, highly soluble and easy to purify and crystallize, the sperm GAPDHS protein becomes highly insoluble, slowly migrating in the gel, and numerous attempts to determine the crystal structure of the whole protein failed due to its properties [9-11]. Its crystal structure without the N-terminal part was found and shows high similarity to the somatic enzyme. As this glycolytic enzyme became a promising target for male non-hormonal contraception long before it was known that the spermatozoa possess the product from the separate gene [7], the structure of the complete protein and its difference from the somatic isoform is crucial for efficient drug design [12].

In mature sperm cells, energy metabolism enzymes are spatially separated, with mitochondria located in the midpiece and glycolytic enzymes in the principal piece of the tail [13]. Previous studies of GAPDHS revealed its localization in the principal piece of the sperm tail [14,15]. Notably, N-terminal polyproline extension has been proposed to facilitate an association and tight binding of the protein to the fibrous sheath in the principal piece [16].

Other proteins, namely at the cell surface, play a role in sperm/oocyte recognition (primary sperm/oocyte binding) [4], and intra-acrosomal proteins participate in the secondary sperm/oocyte binding [17]. The first step in characterization of the cellular functions is identification of the proteins involved. Understanding the physiological role of certain proteins determines their use in further research, diagnostic applications and development of specific treatments.

In our previous work, we tested the effect of selected antibodies on the sperm/egg binding in the swine model. One of the tested antibodies was monoclonal antibody Hs-8. This antibody had no influence on the cell surface sperm proteins and sperm/oocyte binding after incubation with the sperm. On the other hand, when the antibody was present in the medium during sperm and oocyte co-incubation, after acrosome reaction it was bound to the acrosome proteins and prevented the sperm/egg binding. We have confirmed that the antibody binds to the acrosomal proteins that might be involved in the secondary binding of sperm to the egg [5].

In the present work, we identified GAPDHS as a specific antigen for Hs-8 antibody in the sperm cells and confirmed that both Hs-8 and commercial GAPDHS antibodies block the sperm/zona pellucida binding *in vitro*.

## Methods

### Chemicals

Analytical-grade chemicals were utilized. BSA (albumin bovine fraction V, pH 7.0), Immobilon-P membrane, Tween 20, Triton X-100 and Coomassie Brilliant Blue (CBB) R-250 were obtained from Serva (Germany). Gelatin, dithiothreitol (DTT), iodoacetamide, 3-[(3-cholamidopropyl) dimethylammonio]-1-propanesulphonate (CHAPS) were from Sigma (Prague, Czech Republic); thiourea, urea and IPG buffer (pH 3–10) were purchased from Amersham Biosciences (Uppsala, Sweden). Protein standards were from Bio-Rad (Hercules, CA, USA), chemiluminescent substrate (SuperSignal, Pierce) was obtained from Rockford (USA) and VectaShield mounting medium for fluorescence with DAPI H-1200 from Vector Laboratories, (Burlingame, CA, USA).

### Cells

Human ejaculated spermatozoa were obtained from the Iscare IVF Ltd., and Pronatal Ltd., Prague. All sperm donors gave their written informed consent with donating the sperm ejaculates for the purposes of the research project. The study was also approved by the institutional review board at the Institute of Biotechnology. The evaluation of semen density, motility and morphology was carried out in compliance with World Health Organization standards [18]. Boar ejaculates were obtained from the Insemination Station Klimetice (Czech Republic). Mouse spermatozoa were obtained from the proximal fifth region of the left and right cauda epididymis of BALB/c mice (AnLab Ltd., Prague, Czech Republic). All procedures were approved by the Committee for Animal Welfare and Protection.

### Antibodies

Monoclonal antibodies designated Hs-8 and ACR.2 were prepared in our laboratory by immunization of BALB/c mice with human ejaculated sperms. Hyperimmune spleen cells were fused with Sp2/0 myeloma cells. Positive clones were selected by ELISA with human sperm extracts and by indirect immunofluorescence with human spermatozoa. The immunization procedure and hybridoma technology were described in detail by Peknicova et al. [19]. Monoclonal antibody Hs-8 reacted with intra-acrosomal human and boar sperms [5]. Commercial mouse monoclonal antibody (MoAb) IgG1 to recombinant GAPDHS (ab57062, Abcam, UK) was used in verification tests. Monoclonal anti- progesterone (P4) was raised in our laboratory by immunization of mice with P4-BSA conjugate and selected with P4-OVA conjugate. Goat anti-mouse IgG (γ-chain specific) and IgM (μ-chain specific) antibodies conjugated with fluorescein isothiocyanate (FITC) (Sigma, Prague, Czech Republic), goat anti-mouse IgG-PE antibody (Santa Cruz Biotechnology, USA), horseradish peroxidase (HRP)-conjugated goat anti-mouse antibody (GAM/Px) from Bio-Rad (Prague, Czech Republic) were used as secondary antibodies.

### Indirect immunofluorescence and co-localization

Human and boar ejaculated sperms and mouse epididymal spermatozoa were suspended into phosphate-buffered saline (PBS, pH 7.4) for 5 min at 37°C and centrifuged for 15 min at 200× *g*. Next, sperm cells were washed twice and diluted in PBS to a final concentration of 50 × 10^6^ cells ml^−1^. Small drops of the cell suspension were smeared (10 μl) onto glass slides and the remaining spermatozoa were used for protein extraction.

Dried smears were fixed with acetone (10 min, room temperature), rinsed with PBS, and after blocking with bovine serum albumin (1 h, 2% bovine serum albumin in PBS) incubated for 1 h with Hs-8 MoAb (undiluted hybridoma supernatant, immunoglobulin (Ig) concentration < 20 μg ml^−1^) and with mouse monoclonal antibody to recombinant GAPDHS for 60 min at 37°C. For appropriate controls, smears were incubated with nonspecific monoclonal antibody, with the supernatant of myeloma cells, and with the FITC-conjugate only. After washes with PBS, the smears were incubated with secondary antibodies. In case of MoAb Hs-8, the secondary FITC-conjugated goat anti-mouse IgM (diluted 1:128 in PBS) was used and for anti-GAPDHS, FITC-conjugated goat anti-mouse IgG (diluted 1:64 in PBS) was used and incubated for 60 min at 37°C, washed in PBS, rinsed with distilled water and mounted in Vectashield medium. For double immunolabelling experiments, glass slides with the sperm smears were fixed and blocked as described above and incubated with MoAb Hs-8 and FITC-conjugated anti-IgM secondary antibody. The same slides were incubated subsequently with the commercial anti-GAPDHS antibody and goat anti-mouse IgG-PE secondary antibody.

Samples (200 sperm cells per slide) were evaluated and viewed with a Nikon Eclipse E400 fluorescent microscope equipped with 40x Nikon Plan 40/0.65 lenses and photographed with a CCD camera VDS1300 (Vosskühler, Osnabrück, Germany) with the aid of the NIS elements AR imaging software (Laboratory Imaging, Prague, Czech Republic).

### Immunoelectron microscopy

Human fresh ejaculated sperm was separated by SupraSperm System (ORIGIO, Denmark), washed three times in PBS and fixed on ice for 30 min at 0°C in 3% paraformaldehyde and 0.1% glutaraldehyde in Sörensen buffer (SB; 0.1 M sodium/potassium phosphate buffer, pH 7.3), washed twice with SB (10 min each). Cells were then dehydrated in a series of ethanol solutions with increasing concentration of ethanol. Ethanol was then replaced in two steps by LR White resin (Polysciences Inc., Warrington, USA), and the resin was polymerized for two days at +4°C under UV light. After cutting 80 nm sections, nonspecific labelling was blocked by preincubation with 10% normal goat serum (British BioCell International Ltd., Cardiff, UK), 1% BSA and 0.1% Tween 20 in PBS for 30 min at room temperature (RT). For double immunogold labelling experiments, the sections were simultaneously incubated with Hs-8 and commercial anti-GAPDHS primary antibodies, washed three times in PBT (0.005% Tween 20 in PBS), and then incubated with 6 nm gold-conjugated Goat Anti-Mouse IgG (Fcγ fragment specific) and 12 nm gold-conjugated Goat Anti-Mouse IgM (μ chain specific) secondary antibodies (Jackson Immuno Research Laboratories, inc., USA), washed again twice in PBT, then twice in bi-distilled water, and air dried. Finally, sections were contrasted with a saturated solution of uranyl acetate in water (4 min) and observed in electron microscope Morgagni 268 (FEI, Czech Republic) operated at 80 kV. Control incubations without primary antibodies proved that the signal was highly specific and that there was no cross-reactivity in case of multiple labelling.

### Isoelectric focusing, SDS-PAGE and Western blotting

Washed spermatozoa were diluted in PBS, centrifuged at 10000x *g* for 5 min, and extraction buffer was added to the sperm pellet (100 μl of the extraction buffer per 10 × 10^7^ cells): SDS (2% m/v SDS (sodium dodecyl sulphate), 1% v/v glycerol, 50 mM Tris buffer titrated with HCl to pH 6.8) or RHB (rehydration buffer: 7 M urea, 2 M thiourea, 4% CHAPS,1% Triton X-100, 20 mM Tris). SDS extracts were vortexed, boiled in water bath for 3 min, cooled to 4°C and centrifuged (23,000x g, 5 min, 4°C). RHB extracts were incubated for 1 h at RT and centrifuged (23,000x *g*, 5 min, RT). The solubilized samples were divided into aliquots and stored at −80°C for electrophoresis and subsequent analysis. The pure GAPDHS recombinant protein (PO1) from Abnova (Oxford, UK) was directly used for the analysis.

The sperm samples were mixed with RHB, 2% (v/v) IPG buffer (3–10), 1% DTT and 0.005% bromophenol blue (added to the final concentration) and incubated for 1 h at RT. The solubilized proteins (200 μg of proteins in total volume 180 μl) were placed onto 7-cm, pI range 3–10, linear strips and rehydrated overnight (according to manufacturer’s instructions). Strips were focused at RT. For 2D electrophoresis, 12% sodium dodecyl sulphate-polyacrylamide gel electrophoresis (SDS-PAGE) was used.

SDS-PAGE was carried out in 12% slab gels. The protein samples were mixed with reducing SDS sample buffer (50 mM Tris buffer titrated with HCl to pH 6.8, 1% v/v glycerol, 2% m/v SDS, 5% v/v 2-mercaptoethanol, 0.002% m/v bromophenol blue) and boiled for 3 min. Samples of proteins of the sperm extract (total quantity 25 μg) and recombinant protein GAPDHS (5 μg) were applied to the wells. Electrophoretic separation was carried out at constant current 16 mA for each gel in Tris-glycine electrophoretic buffer, pH 8.3 (25 mM Tris, 192 mM glycine), with 0.1% m/v SDS at 4°C. The relative molecular masses of the separated proteins were estimated using prestained Precision Plus Protein Standards run in parallel.

Further, the proteins were transferred onto Immobilon-P membrane for immunodetection. Electroblotting was carried out for 1.5 h at 500 mA and 4°C in TRIS-glycine buffer (pH 9.6) with 20% (v/v) methanol. The membrane was blocked with 5% (w/v) gelatin in PBS-T (0.05% Tween 20 in PBS) at 4°C overnight. After washing with PBS-T, the membrane was incubated with supernatant MoAb Hs-8 (1:15 dilution in 1% gelatin-PBS-T) or anti-GAPDHS MoAb (0.1 μg/μl) at RT for 1 h. Following a washing step, incubation with (GAM/Px) (diluted 1:3000 in 1% gelatin-PBS-T) was peformed for 1 h at RT. After washing, the membrane was developed in the dark with chemiluminescent substrate (SuperSignal) to visualize the corresponding bands.

### CBB staining and sequence analysis

Gels intended for mass spectrometric analysis were stained with Coomassie Brilliant Blue (CBB) for visualization of all separated proteins. After SDS-PAGE, the gels were incubated at RT in a solution containing CBB (0.25% CBB R-250, 7% CH3COOH, 50% ethanol) for 1 h. After incubation with CBB, the gels were destained in 35% ethanol with 10% CH3COOH until the background disappeared and the separated proteins were clearly visible.

The mass of individual peptides obtained after tryptic digestion of Hs-8-detected protein was determined by the MALDI method. Mass spectra of peptides were measured using a MALDI-Time-of-Flight (MALDI-TOF) mass spectrometer, a peptide map was established and mass spectra were searched against the database using Profound software. Mass spectrometer BIFLEX II (Bruker-Franzen, Bremen, Germany) was equipped with a nitrogen laser (337 nm) and a gridless delayed extraction ion source. Ion acceleration voltage was 19 kV and the reflectron voltage was set to 20 kV. The spectrum was calibrated internally using the monoisotopic [M+ H]^+^ ions of trypsin autoproteolytic products. A saturated solution of α-cyano-4-hydroxy-cinnamic acid in 50% ACN/0.2% trifluoroacetic acid was used as a MALDI matrix. One microliter of matrix solution was mixed with 1 ml of the sample on the target and the droplet was allowed to dry at ambient temperature.

### Isolation and culture of porcine oocytes

Porcine oocytes were recovered from fresh ovaries about 3 h after slaughter by puncturing and aspirating of 3 to 5 mm follicles. Oocytes were collected in BSA-PBS medium, placed in MPM (modified Parker medium) under paraffin oil, and incubated for 48 h at 37°C under 5% CO_2_ to complete maturation. After culturing, oocytes were transferred in a number of 40 to 50 pieces in 0.5 ml of 3 M DMSO in culture medium for 10 min at 4°C, and the tubes with oocytes were placed into liquid nitrogen vapours until use. On the day of binding assay, the tubes with frozen oocytes were thawed at 37°C, 5% CO_2_. Then the oocytes were washed three times in BSA-PBS, transferred to the drops of medium and overlaid with paraffin oil.

### In vitro sperm-zona pellucida-binding assay

We examined the effect of monoclonal antibodies (Hs-8 and anti GAPDHS) during sperm/oocyte co-incubation. The oocytes (10–20) were transferred to 120 μl droplets containing monoclonal antibodies Hs-8 and GAPDHS, respectively, or monoclonal antibody against progesterone, ACR.2, or MPM medium only (control groups). Capacitated spermatozoa (50 μl) were added to the control and experimental groups of oocytes. After 30 min, co-incubation was stopped by adding 50 μl of 10% NaN_3_. Oocytes with bound spermatozoa were washed twice and fixed in 2.5% paraformaldehyde. After washing, the fixed oocytes were stained with Hoechst 33342 (Sigma, Prague) solution (0.3 mg Hoechst /10 ml BSA-PBS), rinsed twice and mounted in a very small droplet of 50% glycerol in PBS (pH 9.0), and Hoechst-labelled spermatozoa attached per oocyte were counted under a fluorescent microscope.

### Statistical analysis

Experimental data were analysed and plotted using STATISTICA 6.0. and GraphPad Prism 5.04. Twenty oocytes were analysed per each control and experimental group (N = 20) in each experiment; the total number of analysed oocytes was 100. Sperm samples were obtained from two boars. The differences in the number of bound sperm cells among control and experimental groups were analysed by Kruskal-Wallis test, the post-hoc analysis was performed by Dunn’s multiple comparison test. The p value equal to or lower than 0.05 was considered to be significant, *p value ≤0.05 (**p ≤0.01 and ***p ≤0.001).

## Results

### Indirect immunofluorescence and localization of Hs-8 protein on mammalian sperm

**Figure 1:**
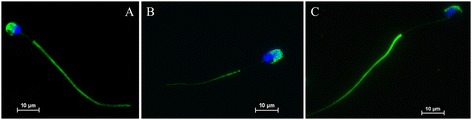
**Cellular localization of the Hs-8 antigen in mammalian spermatozoa.** IF of human **(A)**, boar **(B)** and mouse **(C)** spermatozoa with Hs-8 monoclonal antibody (*green),* nuclear staining (*blue*). Bar: 10 μm.

We analysed the interaction of the mouse monoclonal Hs-8 antibody with human, boar and mouse sperm cells. The staining pattern was similar in all these species. The signal was present in the acrosomal part of the sperm head and in the principal piece of the sperm flagellum, while it was absent in the periacrosomal part and midpiece (Figures 1A,B,C). The recognized epitope on the sperm cells seems to be conserved in all the studied species.

### Biochemical characterization of the Hs-8 target antigen

**Figure 2:**
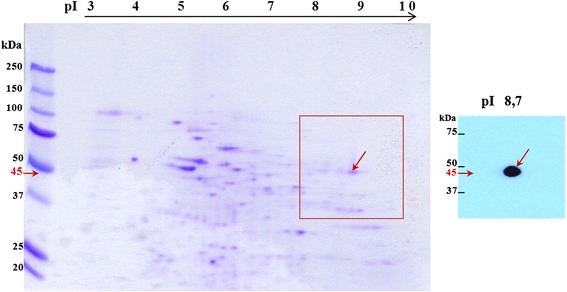
**Localization of the Hs-8 target antigen on the two-dimensional map of human sperm proteins.** 2D-PAGE (12%), Western blotting and immunodetection of human sperm extract with MoAb Hs-8, which labelled a protein of 45 kDa.

To identify and characterize the antigen that was recognized by MoAb Hs-8, the lysate of human sperm cells was separated with 2D gel electrophoresis followed by protein detection. The signal was obtained at basic pI 8 and 50 kDa (Figure 2). Next, the corresponding protein dot was excised from the polyacrylamide gel and sequenced. Sequence analysis identified the target protein of the MoAb Hs-8 as GAPDHS (glyceraldehyde 3-phosphate dehydrohenase-spermatogenic), nominal mass (Mr): 44.83 kDa; pI value: 8.7. The number of peptides searched was 24 and the number of those that matched the identified protein was 18. Total sequence coverage was 48% and estimated Z-score: 2.32.

### The specificity of Hs-8 antibody and commercial mouse anti-GAPDHS antibody to GAPDHS protein

**Figure 3:**
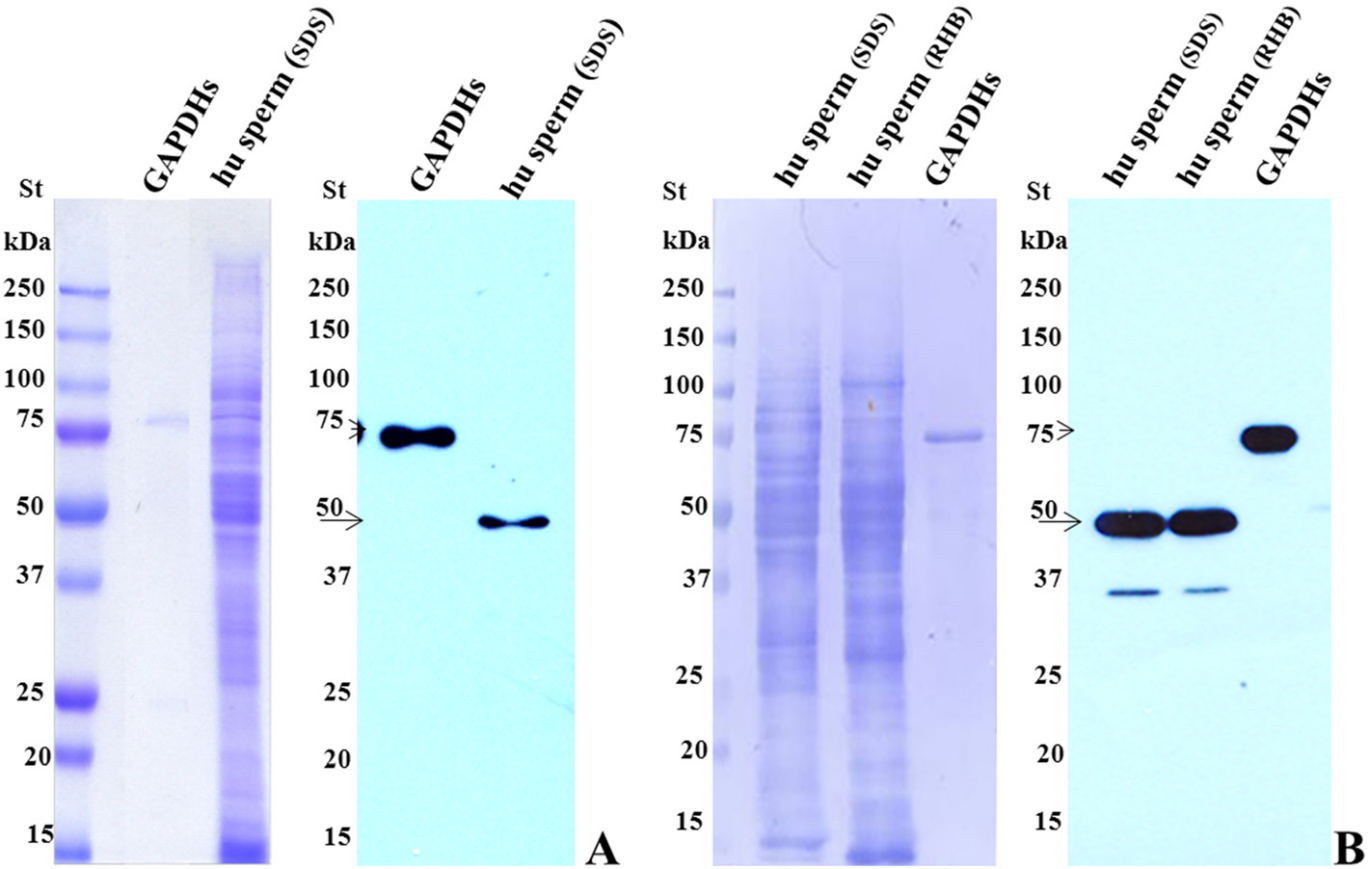
**Biochemical confirmation of the Hs-8 target specificity.** Immunodetection of human sperm extracts and purified recombinant GAPDHS-GST tagged protein with MoAb Hs-8 **(A)** and with commercial mouse monoclonal antibody against GAPDHS **(B)**. Arrows indicate the GAPDH protein labelled with anti-GAPDHS antibody. St – protein standard, hu sperm – human sperm cell lysate, GAPDHs – recombinant purified GAPDHS protein, hu sperm (SDS) – SDS extract of human sperm cells, hu sperm (RHB) – human sperm cell proteins extracted with RHB buffer.

**Figure 4:**
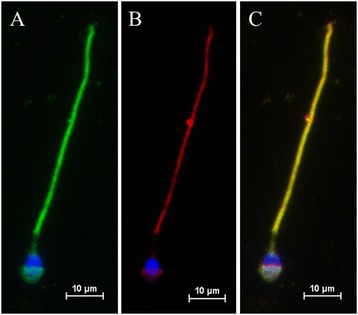
**Co-localization of Hs-8 and anti-GAPDHS antigens in the human sperm cells. A**. Hs-8 staining of the sperm cell. Indirect immunofluorescence of human spermatozoa with MoAb Hs-8 (green). **B**. Cellular localization of GAPDHS protein detected with anti-GAPDHS antibody (red). **C**. Merge. Yellow colour represents co-localization of the Hs-8 antigen with GAPDHS signal in the sperm head and tail. Bar: 10 μm.

To confirm that the antigen recognized by Hs-8 is indeed the GAPDHS protein, commercial antibody anti-GAPDHS and recombinant GAPDHS protein conjugated with GST tag were used for analysis. Both Hs-8 (Figure 3A) and anti-GAPDHS (Figure 3B) antibodies labelled bands of the same molecular weight (50 kDa) in immunoblot analysis. At the same time, recombinant protein with MW of 75 kDa was recognized by both commercial anti-GAPDHS and Hs-8 (Figures 3A and B, respectively). Next, we looked whether the data from biochemical analysis could be confirmed by indirect immunofluorescense. Both home-made Hs-8 and commercial anti-GAPDHS are mouse antibodies, but of different isotypes, and double staining could be performed using differently conjugated secondary antibodies against either IgG or IgM. Double immunolabelling with both antibodies demonstrated strong co-localization of the signal in both principal piece and apical part of the sperm head, suggesting that the antibodies recognize the same antigen (Figure 4).

### Ultrastructural localization of the GAPDHS protein

**Figure 5:**
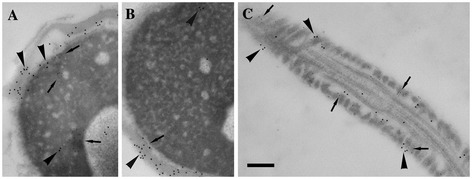
**Ultrastructural localization of Hs-8 and GAPDHS antigens.** Immunogold labelling of the sections of the sperm head **(A, B)** and principal piece of the tail **(C)** with Hs-8 (12 nm gold particles, arrowheads) and anti-GAPDHS (6 nm gold particles, arrows) antibodies. The signal after staining with either antibody showed a similar pattern and the gold particles appeared in the fibrous sheath of the principal piece in the tail **(C)** and acrosomal part of the sperm head **(A, B)**. Bar: 200 nm.

Ultrastructural analysis of double immunogold labelling with both antibodies was performed with electron microscopy for precise localization of GAPDHS in the sperm head. It showed that the Hs-8 antigen (12 nm gold particles, arrowheads) and GAPDHS protein (6 nm gold particles, arrows) are localized in close proximity in the nuclear and acrosomal regions of the sperm head (Figures 5A and B, respectively) and in the sperm tail (Figure 5C).

### Effect of monoclonal antibodies Hs-8 and GAPDHS on sperm/zona pellucida-binding

**Figure 6:**
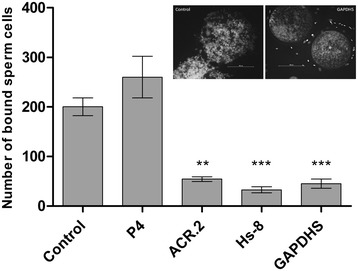
**Boar sperm-zona pellucida binding in the presence of Hs-8 and anti-GAPDHS antibodies during sperm-oocyte co-incubation.** The number of bound sperm cells on the oocyte after co-incubation with no antibody (Control), and various antibodies: anti-progesterone (P4), acrosin (ACR.2), Hs-8 and anti-GAPDHS (GAPDHS). Two representative images showing sperm oocyte binding with anti-GAPDHS (right side) and no antibody (left side) are displayed in the chart area. * - p value ≤0.05, ** - p ≤0.01 and *** - p ≤0.001).

During co-incubation of capacitated spermatozoa with oocytes in the presence of monoclonal antibodies Hs-8 and GAPDHS, and ACR.2 as positive control, the sperm binding was strongly reduced. The presence of Hs-8 and GAPDHS decreased the number of spermatozoa to 18% and 21%, respectively, compared to the control group (without antibody). In the positive control with monoclonal antibody ACR.2 (against acrosin), the number of bound spermatozoa was reduced by 25%, see also Figure 6.

## Discussion

Among useful tools for studying sperm cell proteins are monoclonal antibodies. If the antibody detects a protein with important functions, it can be used in diagnostics [20-22]. In our laboratory, we prepared a panel of monoclonal antibodies against intra-acrosomal proteins that can be used for determination of sperm quality by assessing the acrosomal status. Using these antibodies, we assessed the quality of mouse spermatozoa [23-25]. We also used monoclonal antibodies against sperm proteins for evaluation of the sperm quality in experimentally induced pathology in mice using endocrine disruptors or anti-androgens [26,5,27] and in humans for evaluation of sperm pathology [28]. Monoclonal antibody Hs-8 against human intra-acrosomal sperm protein is part of this panel. This antibody was used as a tool for evaluation of the human sperm quality and reproductive potential after intra-cytoplasmic sperm injection of various human semen samples [29,28] and is part of the commercial SpermFlow Kit (Exbio, Czech Republic). For this reason, we wanted to further characterize and identify its target protein.

Biochemical and sequence analysis revealed that this sperm antigen is the testes-specific glycolytic enzyme GAPDHS. Slower migration of the protein band at ca 50 kDa instead of the calculated 45 kDa in SDS-PAGE was shown to be due to the proline-rich N-terminal part of the protein and indirectly confirms that GAPDHS is the target antigen for Hs-8 antibody. Indeed, Hs-8 antibody readily interacted with purified GAPDHS-GST on western blot in a similar way as commercial anti-GAPDHS antibody. Another proof of the Hs-8 specificity was indirect immunofluorescent and immunogold labelling of the sperm cells with anti-GAPDHS and Hs-8 antibodies. In both cases, the signal appeared in the principal piece and acrosomal part of the sperm cells.

As the GAPDHS presence in the principal piece of the sperm tail was in line with previous studies, GAPDHS localization to the acrosomal part of the sperm head was surprising. Nevertheless, there was already some evidence that at least in boar, GAPDHS is localized not only in the principal piece of the sperm flagellum, but also in the acrosomal part [30]. Moreover, GAPDHS protein was found in the protein pool from the human sperm cell nucleus [31]. This supports the idea that GAPDHS, similarly as its somatic isoform GAPDH, might play some additional function in the sperm cells. It is worth noting that bacterial glycolytic enzyme glyceraldehyde 3-phosphate dehydrogenase (GapA-1) might be involved in adhesion of *Neisseria meningitidis* to human cells [32].

In our previous work, we already tested the effect of Hs-8 antibody on the sperm/egg binding. This antibody had no influence on the cell surface sperm proteins and sperm/oocyte binding after pre-incubation with the sperm prior to the binding assay. However, when the antibody was present in the medium during sperm and oocyte co-incubation, it prevented the sperm/egg binding. It seems that Hs-8 binds to the acrosomal proteins after acrosome reaction and blocks the secondary binding of sperm to the egg [5]. In this study, we tested the effect of both Hs-8 and GAPDHS, while anti-P4 (anti-progesterone) and ACR.2 (anti-acrosin) were used as negative and positive controls, respectively. There was a four- to five-fold decrease in the number of bound sperm cells to the oocyte when ACR.2, Hs-8, or anti-GAPDHS antibodies were present in the incubation medium. Anti-P4 had no effect on the sperm/oocyte binding. The outcome of the *in vitro* sperm-zona binding assay suggests involvement of the GAPDHS protein in the secondary sperm/zona pellucida binding.

Still, further analysis of the exact mechanism of GAPDHS interaction with the oocyte and the finding of its interacting partner are needed in the future to confirm that the GAPDHS enzyme plays a role in mammalian gamete interaction.

## Conclusions

To sum up, we characterized the Hs-8 protein and identified it as the intra-acrosomal sperm protein GAPDHS. We have also found that its probable role in the sperm head is the secondary binding of sperm to the egg.
